# Supplementary material for: An efficient real-time signal processing method for satellite laser ranging
Source: PLoS One. 2024 Dec 20;19(12):e0315375. doi: 10.1371/journal.pone.0315375 (PMC11661637; doi:10.1371/journal.pone.0315375)
Supplement: S1 File — (DOCX) [file pone.0315375.s001.docx]

%The annotations are in Chinese，Please run the following code in MATLAB.

%读取预报数据

report_data = load('D:\desktop\SLR_data\090613etalon1report__1000.txt');%将数据以矩阵的形式保存

column4 = report_data(:, 4); % 第四列

x_report= 0:1:100;

y_report=column4;%原始数据设定为0-100s，及其对应的预报值

% 新的x轴坐标点，间隔为0.001s

x_interp = 0:0.001:100; % 从0到4，间隔为0.001s

%%插值

% 使用interp1函数进行线性插值

c_y = interp1(x_report, y_report, x_interp, 'linear');%y_interp是数组

% plot(x_interp, c_y, 'k.', 'DisplayName', 'Data');

% hold on;

% plot(x_report, y_report, 'o');

% % legend('已知点', '插值结果');

% xlabel('x轴');

% ylabel('y轴');

% title('插值结果');

%读取实测数据,注意换算一下起始时间

reality_data = load('D:\desktop\SLR_data\090613etalon1tb4_1000.txt');%将数据以矩阵的形式保存

time_h=reality_data(:, 1);

time_m=reality_data(:, 2);

time_s1=reality_data(:, 3);

start_h=15;%数据的开始时刻

start_m=39;

start_s=59;

o_y=reality_data(:, 10);%观测值

%秒，保留三位小数,经过测试，无相邻的数相同

factor = 10^3; % 乘以10^3，这样小数点就向右移动3位

time_s2 = round(time_s1 * factor) / factor;

time_t=time_h*3600+time_m*60+time_s2;

t_mum=(time_t-start_h*3600-start_m*60-start_s);%将数据时间换算到0-100

num=0;%替换的点数

% 生成随机变量

random_noise =-1.35+2.7*rand(size(c_y));%在±1.35之间

random_noise(random_noise>=0.05) = random_noise(random_noise >=0.05)+0.15;%在[0.2 1.5]之间

random_noise(random_noise< 0.05) = random_noise(random_noise<=0.05)-0.15;%在[-1.5 -0.1]之间

result_y = c_y+ random_noise;%生成噪声组，然后把观测数据插入。

% 遍历 B 组的 x 坐标

for i = 1:length(t_mum)

% 查找对应的位置

idx = find(abs(x_interp - t_mum(i)) <0.0001);%找到两组坐标中相同的x（matlab中只能小于一个很小的点，来代表两者相等）

% 如果找到了对应位置，则替换对应的 y 坐标

if ~isempty(idx)

result_y(idx) = o_y(i);

num=num+1;

end

end

% 显示结果

% disp(result_y);

ocy=(result_y-c_y)*10^6;%换算成ps

%

%

% idx = find(abs(ocy > 1500000));%好看图，正式处理的时候，不要这两个

% ocy(idx) = -1500000;

figure;

plot(x_interp(abs(ocy) < 200000), ocy(abs(ocy) <200000), 'k.', 'DisplayName', 'Data');

xlabel('Time (s)');

ylabel('Residual (ps)');

% title('Residual vs Time');

legend('Location', 'best');

grid on;

set(gca, 'FontSize', 15);%设置图像字体大小

%开始模拟读取数据并且处理数据

F=1000;%频率

t=100;%时间

time = 0:(1/F):t;%时间0-t秒

y_max=200000;%残差范围200000ps

max_size = length(time);

x_sorted=zeros(1, max_size);%预先分配比不预先分配快了1s

y_sorted=zeros(1, max_size);

x_sorted1=zeros(1, max_size);

y_sorted1=zeros(1, max_size);

x_sorted11=zeros(1, length(time));%两次GRAZ

y_sorted11=zeros(1, length(time));

x_sorted22=zeros(1, length(time));

y_sorted22=zeros(1, length(time));

x_sorted33=zeros(1, length(time));%GRAZ+多项式拟合

y_sorted33=zeros(1, length(time));

x_sorted44=zeros(1, length(time));

y_sorted44=zeros(1, length(time));

counter333=0;%记录符合两次GRAZ的计数器

x_GP=zeros(1, length(time));

y_GP=zeros(1, length(time));

n33=1000;%与前n个点进行比较

m33=3;

td33=500;%阈值

n44=30;%第二次前n个点

counter33=0;%计数符合一次GRAZ的

counter44=0;%计数器

y_30=[];%存放预处理数据

x_30=[];

%两次GRAZ

n11=1000;%与前n个点进行比较

m11=3;

td11=1000;%阈值100ps

n22=300;%第二次GRAZ与前n个点进行比较

m22=3;

td22=500;%阈值100ps

counter11=0;%记录第一次graz的计数器

counter222=0;%记录符合两次GRAZ的计数器

x_G=zeros(1, length(time));

y_G=zeros(1, length(time));

counter22=0;

% GRAZ+直方图，预先分配计数器

counter3=0;

show_rate=1;%显示比例

draw_interval=10;

x_GH=[];

y_GH=[];

%GRAZ+直方图

n_n=0;%噪声点数量

n_s=0;%信号点数量

n1=1000;%与前n个点进行比较

m1=3;

td1=1000;%阈值

counter1=0;%计数符合一次GRAZ的

figure;%画图

grid_size = 2;

grid_y_length = 1000;%方格%如果点全都按照均匀分布，经过计算一个直方图，只有1.25个点。对应精度7.5cm

grid_x_length = grid_size;

move_y_size=grid_y_length/2;

move_x_size=0.5;

grid_data_num = zeros(1, ceil((2*y_max-grid_y_length)/move_y_size)+1);%在每个grid_size，移动的格子中数据个数

grid_data_polyfit=cell(2,ceil((t-grid_size)/move_x_size)+1);%每个grid_size保留一个格子，记录t时间保留的格子数量，用作拟合。

% grid_data_polyfit可以预先设置为存放所有的有效数据

T_count=1;%计数器记录第几个时刻(第一个格子被舍弃掉了)

%T_count_s=0;%计数器记录第几个有效时刻

% 遍历每个0.1的格子

first_GRAZ_p = [];%graz第一个处理的信号的位置

first_GRAZ_num= [];

a=F*grid_size;%一个直方图最多多少个点

tic;

for i = 1:length(time)

x_sorted(i)=time(i);

y_sorted(i)=(result_y(i)-c_y(i))*10^6;%OC值

if rand(1)<show_rate %显示比例

if i>n1%GRAZ算法

% for j = 1001:i

% 计算与前n1个点的差值

differences = abs(y_sorted(i) - y_sorted(i-n1:i-1));

% 统计差值小于td1的数量

count = sum(differences < td1);

% 如果有超过三个差值小于td1，则将该点显示

if count >=3%判断该点是否满足信号识别算法

counter1=counter1+1;

x_sorted1(counter1)=x_sorted(i);

y_sorted1(counter1)=y_sorted(i);

% ylim([-y_max y_max]);

% plot(x_sorted1(counter1), y_sorted1(counter1), 'k.', 'DisplayName', 'Data');

% hold on;

% if counter3 >= draw_interval

% drawnow;

% hold on;

% counter3 = 0; % 重置计数器

% end

if isempty(first_GRAZ_p)

first_GRAZ_p = x_sorted1(counter1); % 以第一个GRAZ的点为0点

end

if isempty(first_GRAZ_num)

first_GRAZ_num = i; % 第一个GRAZ点的i值

end

%直方图算法横移的策略：以GRAZ处理的第一个点的横坐标为开始，后面每0.1s（i-first_GRAZ_num),grid_size*F）移动一次

end%GRAZ结束

if counter1>1&&mod((i-first_GRAZ_num),move_x_size*F)==0%每move_x_size移动一次（0.5s）

% (直方图移动条件不应该嵌套在if count > 3条件内，应该是无条件移动的)

T_count=T_count+1;

% grid_x_start = (T_count-1)*grid_size+first_GRAZ_p;

% grid_x_end = grid_x_start+ grid_x_length;

% grid_x_end=x_sorted1(counter1);

% grid_x_start=x_sorted1(counter1-4);

grid_x_end=(T_count-1)*move_x_size+first_GRAZ_p;

grid_x_start=grid_x_end-grid_x_length;

count2=0;%计数器，记录第几个格子

for y = -y_max:move_y_size:y_max-grid_y_length%从下往上

grid_y_start = y;

grid_y_end = y+grid_y_length;

% 记录格子中包含的点的数量

count2=count2+1;

if counter1<=a%如果GRAZ识别的数小于a，就从1开始，否则从counter1-a开始，这样保证了在直方图附近的点进行选择，减少了运算量。

points_idx_1 = x_sorted1(1:counter1) >= grid_x_start & x_sorted1(1:counter1) <= grid_x_end & y_sorted1 (1:counter1)>= grid_y_start & y_sorted1(1:counter1) <= grid_y_end;

else

points_idx_1 = x_sorted1(counter1-a:counter1) >= grid_x_start & x_sorted1(counter1-a:counter1) <= grid_x_end & y_sorted1 (counter1-a:counter1)>= grid_y_start & y_sorted1(counter1-a:counter1) <= grid_y_end;

end

% % 记录格子中包含的点的数量

grid_data_num(count2) = sum(points_idx_1);

end

[points_in_grid,grid_index]=max(grid_data_num);%记录最大的格子数量和格子位置

grid_y_start=-y_max+(grid_index-1)*move_y_size;

grid_y_end=grid_y_start+grid_y_length;

% 处理点数量为0的情况

% if points_in_grid == 0

% continue;

% end

%

% 处理点数量超过2的情况

%拟合曲线

if T_count>1 && mod(T_count-1,40)==0 %每过50个格子，也就是25s

if all(cellfun(@isempty, grid_data_polyfit(1,T_count-40:T_count-1)))%判断前50个格子包含的有效数组个数是否为空

flag=0;%标志符，0就是没有拟合曲线

else

flag=1;%标志符，1就是有拟合曲线

x_selected_cells = grid_data_polyfit(1,T_count-40:T_count-1);%选择50个格子

x_non_empty_cells = x_selected_cells(~cellfun(@isempty, x_selected_cells));%过滤出非空元素

y_selected_cells = grid_data_polyfit(2,T_count-40:T_count-1);

y_non_empty_cells = y_selected_cells(~cellfun(@isempty, y_selected_cells));

x_polyfit = cat(2, x_non_empty_cells{:});%合并非空元素到一个新的数组中

y_polyfit = cat(2, y_non_empty_cells{:});

p = polyfit(x_polyfit, y_polyfit, 1);

end

end

if points_in_grid >5%有效数据

% T_count_s=T_count_s+1;

% 绘制格子所有数据

if flag==1

points_idx = x_sorted1 >= grid_x_start & x_sorted1 <= grid_x_end & y_sorted1 >= grid_y_start & y_sorted1 <= grid_y_end;

grid_data_polyfit{1,T_count}=x_sorted1(points_idx);%记录符合要求的最大格子中的数据

grid_data_polyfit{2,T_count}=y_sorted1(points_idx);

x_histogram=x_sorted1(points_idx);%提取方格中的数据

y_histogram=y_sorted1(points_idx);

% 计算每个点到直线的距离

distances = abs(p(1) * x_histogram - y_histogram + p(2)) / sqrt(p(1)^2 + 1);%p(1)斜率，p(2)截距

% 判断距离在move_size以内的点

valid_points_idx = (distances <= 1/2*move_y_size);

% 绘制格子中有效数据点

if any(valid_points_idx)

x_GH=[x_GH x_histogram(valid_points_idx)];

y_GH=[y_GH y_histogram(valid_points_idx)];

xlabel('Time (s)');

ylabel('Residual (ps)');

ylim([-y_max y_max]);

plot(x_histogram(valid_points_idx), y_histogram(valid_points_idx), 'k.', 'DisplayName', 'Data');

hold on;

counter3 = counter3 + 1;

if counter3 >= draw_interval

drawnow;

hold on;

counter3 = 0; % 重置计数器

end

% is_in_signal = ismember(x_histogram(valid_points_idx), signal_x);%判断信号识别算法标记的点是否是自动生成的信号点

% num_in_signal = sum(is_in_signal);

% num_not_in_signal = numel(x_histogram(valid_points_idx)) - num_in_signal;

% n_s=n_s+num_in_signal;

% n_n=n_n+num_not_in_signal;

end

else

valid_points_idx = (x_sorted1 >= grid_x_start & x_sorted1 <= grid_x_end & y_sorted1 >= grid_y_start & y_sorted1 <= grid_y_end);

grid_data_polyfit{1,T_count}=x_sorted1(valid_points_idx);%记录符合要求的最大格子中的数据

grid_data_polyfit{2,T_count}=y_sorted1(valid_points_idx);

x_GH=[x_GH x_sorted1(valid_points_idx)];

y_GH=[y_GH y_sorted1(valid_points_idx)];

xlabel('Time (s)');

ylabel('Residual (ps)');

ylim([-y_max y_max]);

plot(x_sorted1(valid_points_idx), y_sorted1(valid_points_idx), 'k.', 'DisplayName' ,'Data');

hold on;

counter3 = counter3 + 1;

if counter3 >= draw_interval

drawnow;

hold on;

counter3 = 0; % 重置计数器

end

% is_in_signal = ismember(x_sorted1(valid_points_idx), signal_x);%判断信号识别算法标记的点是否是自动生成的信号点

% num_in_signal = sum(is_in_signal);

% num_not_in_signal = numel(x_sorted1(valid_points_idx)) - num_in_signal;

% n_s=n_s+num_in_signal;

% n_n=n_n+num_not_in_signal;

end

end

% 处理点数量在0和2之间的情况

if points_in_grid<=5

if flag==1

points_idx = x_sorted1 >= grid_x_start & x_sorted1 <= grid_x_end;

% points_idx = x_sorted1 >= grid_x_start & x_sorted1 <= grid_x_end & y_sorted1 >= grid_y_start & y_sorted1 <= grid_y_end;

x_histogram=x_sorted1(points_idx);%提取方格中的数据

y_histogram=y_sorted1(points_idx);

% 计算每个点到直线的距离

distances = abs(p(1) * x_histogram - y_histogram + p(2)) / sqrt(p(1)^2 + 1);%p(1)斜率，p(2)截距

% 判断距离在move_size以内的点

valid_points_idx = distances <= 1/2*move_y_size;

% 绘制格子中有效数据点

if any(valid_points_idx)

x_GH=[x_GH x_histogram(valid_points_idx)];

y_GH=[y_GH y_histogram(valid_points_idx)];

xlabel('Time (s)');

ylabel('Residual (ps)');

ylim([-y_max y_max]);

plot(x_histogram(valid_points_idx), y_histogram(valid_points_idx), 'k.', 'DisplayName', 'Data');

hold on;

counter3 = counter3 + 1;

if counter3 >= draw_interval

drawnow;

hold on;

counter3 = 0; % 重置计数器

end

% is_in_signal = ismember(x_histogram(valid_points_idx), signal_x);%判断信号识别算法标记的点是否是自动生成的信号点

% num_in_signal = sum(is_in_signal);

% num_not_in_signal = numel(x_histogram(valid_points_idx)) - num_in_signal;

% n_s=n_s+num_in_signal;

% n_n=n_n+num_not_in_signal;

end

end

end

end%直方图

end%GRAZ开始

end

end

set(gca, 'FontSize', 15);%设置图像字体大小

elapsed_time = toc;

disp(['本算法执行时间为：', num2str(elapsed_time), ' 秒']);

% figure;

% plot(x_GH, y_GH, 'b.', 'DisplayName', 'Data');

% ylim([-y_max y_max]); % 设置y轴范围

% xlim([0 t]);

%两次GRAZ

tic;

figure;

for i = 1:length(time)

x_sorted11(i)=time(i);

y_sorted11(i)=(result_y(i)-c_y(i))*10^6;%OC值

if rand(1)<show_rate %显示比例

if i>n11%GRAZ算法

differences = abs(y_sorted11(i) - y_sorted11(i-1000:i-1));

% 统计差值小于100的数量

count = sum(differences < td11);

if count >=m11%判断该点是否满足信号识别算法

counter11=counter11+1;

x_sorted22(counter11)=x_sorted11(i);

y_sorted22(counter11)=y_sorted11(i);

if counter11>n22 %第二次GRAZ

differences1 = abs(y_sorted22(counter11) - y_sorted22(counter11-300:counter11-1));

% 统计差值小于100的数量

count1 = sum(differences1 < td22);

% 如果有超过三个差值小于1000，则将该点显示

if count1 >=m22%判断该点是否满足信号识别算法

counter222=counter222+1;

x_G(counter222)=x_sorted22(counter11);

y_G(counter222)=y_sorted22(counter11);

xlabel('Time (s)');

ylabel('Residual (ps)');

ylim([-y_max y_max]); % 设置y轴范围

xlim([0 t]);

plot(x_sorted22(counter11), y_sorted22(counter11), 'k.', 'DisplayName', 'Data');

hold on;

% scatter(x_sorted1(counter1), y_sorted1(counter1), 'k.', 'DisplayName', 'Data');

% 实时绘制图形

counter22 = counter22 + 1;

if counter22 >= draw_interval

drawnow;

hold on;

counter22 = 0; % 重置计数器

end

end

end

end

%pause(0.01); % 等待0.01秒，可以根据需要调整

end

end

end

set(gca, 'FontSize', 15);%设置图像字体大小

elapsed_time = toc;

disp(['两次GRAZ执行时间为：', num2str(elapsed_time), ' 秒']);

x_G = x_G(1:counter222);

y_G = y_G(1:counter222);% 缩小数组大小到实际使用大小

% figure;

% plot(x_G, y_G, 'b.', 'DisplayName', 'Data');

% ylim([-y_max y_max]); % 设置y轴范围

% xlim([0 t]);

tic;

figure;%GRAZ+多项式

for i = 1:length(time)

x_sorted33(i)=time(i);

y_sorted33(i)=(result_y(i)-c_y(i))*10^6;%OC值

if rand(1)<show_rate %显示比例

if i>n33%GRAZ算法

differences = abs(y_sorted33(i) - y_sorted33(i-n33:i-1));

% 统计差值小于td33的数量

count = sum(differences < td33);

if count >=3%判断该点是否满足信号识别算法

counter33=counter33+1;

x_sorted44(counter33)=x_sorted33(i);

y_sorted44(counter33)=y_sorted33(i);

if counter33>n44

y_30=y_sorted44(counter33-n44:counter33-1);%预处理数据

x_30=x_sorted44(counter33-n44:counter33-1);

% 计算均值和标准差

mean_val = mean(y_30);

std_val = std(y_30,1);%除n-1的样本标准差

% 得出拟合数据

y_polyfit = y_30(abs(y_30 - mean_val) < 3 * std_val);

x_polyfit = x_30(abs(y_30 - mean_val) < 3 * std_val);

n_polyfit=length(y_polyfit);%拟合点的数量

p3 = polyfit(x_polyfit, y_polyfit, 1);

s = sqrt(sum((y_polyfit - polyval(p3, x_polyfit)).^2) / (n_polyfit - 1));%标准差

if abs(y_sorted44(counter33)-polyval(p3, x_sorted44(counter33)))<2.5*s

counter333=counter333+1;

x_GP(counter333)=x_sorted44(counter33);

y_GP(counter333)=y_sorted44(counter33);

xlabel('Time (s)');

ylabel('Residual (ps)');

ylim([-y_max y_max]); % 设置y轴范围

xlim([0 t]);

plot(x_sorted44(counter33), y_sorted44(counter33), 'k.', 'DisplayName', 'Data');

hold on;

counter44 = counter44 + 1;

if counter44 >= draw_interval

drawnow;

hold on;

counter44 = 0; % 重置计数器

end

end%输出当前有效点

end

end%符合一次GRAZ

end%开始GRAZ

end

end

set(gca, 'FontSize', 15);%设置图像字体大小

elapsed_time = toc;

disp(['GRAZ+多项式执行时间为：', num2str(elapsed_time), ' 秒']);

x_GP = x_GP(1:counter333);

y_GP = y_GP(1:counter333);% 缩小数组大小到实际使用大小

% figure;

% plot(x_GP, y_GP, 'b.', 'DisplayName', 'Data');

% ylim([-y_max y_max]); % 设置y轴范围

% xlim([0 t]);
