# Supplementary material for: An efficient real-time signal processing method for satellite laser ranging
Source: PLoS One. 2024 Dec 20;19(12):e0315375. doi: 10.1371/journal.pone.0315375 (PMC11661637; doi:10.1371/journal.pone.0315375)
Supplement: Attachment — Submitted filename: Response to Reviewers.docx [file pone.0315375.s008.docx]

Manuscript Number: PONE-D-24-45347

Title: An Efficient Real-Time Signal Processing Method for Satellite Laser Ranging

Authors: Jinyu Wang, Wei Zhu*, Weiming Gong

Dear editor and reviewers,

Thank you for your letter and comments concerning our manuscript entitled “An Efficient Real-Time Signal Processing Method for Satellite Laser Ranging”. These comments are very helpful in modifying and improving our paper, as well as the important guiding significance to our researches. We have carefully studied these comments and have made corrections which we hope meet with approval. Here's a point-to-point response to your comments.For clarity, the revision in the latest manuscript is printed in red color. The blue text represents the author's explanation.

**Editor:**

**The authors can improve the literature review in the manuscript. The authors need to mention the keyworks on different processing Method for Satellite Laser Ranging. Moreover, provide the tabular comparison of different processing methods/algorithms with the performance of the proposed method.**

Author’s Response: We sincerely thank the editor for their constructive comments regarding the improvement of the literature review. Based on your suggestions, the revised version is as follows:

(Introduction)

Satellite laser ranging (SLR) is a high-precision geodetic method used in many fields, such as geodynamics, geodesy, and astronomy [1-4] SLR obtains the distance between the Earth and the satellite by transmitting laser pulses that accurately measure the time from the ground observation point to the satellite equipped with a corner reflector [5-7]. Currently, with the continuous advancement of kHz satellite laser technology, the volume of echo data has significantly increased [8-11]. However, due to the influence of sky background noise and system noise, a substantial amount of noise is present in the echo signals[12-14]. This issue is further exacerbated in observations of high-orbit satellites and space debris, where the echo signals are notably weaker[15-17].During observations, operators must adjust the laser's position based on whether valid signals are present in the echo data to ensure the laser accurately targets the satellite. Therefore, effective data processing is crucial for optimizing laser ranging observations and ensuring precise satellite tracking.

The manual screen recognition method relies on a human-computer interaction interface, where observers manually filter valid signals based on experience. While it offers strong adaptability and effectively filters noise, it involves extensive manual intervention, has low automation, and results in reduced efficiency. [18-20]. The Poisson filtering algorithm segments data over short time intervals using tilted grids. When the number of echo points within a grid exceeds a predefined threshold and follows a Poisson process, the region is determined to contain valid echo data. This method performs well in practice. However, its detection results are significantly influenced by Poisson statistical filtering, and it lacks strong adaptability in data processing[21-23].To improve the automation of processing data, various histogram methods have been proposed to determine whether the echoes in the box are valid or not by setting the size of the distance window, the moving interval, and the threshold according to the statistical characteristics of the SLR echoes [24]. Graz fast echo recognition algorithm [25-26], is one of the typical algorithms, the algorithm compares the current point with the previous 1,000 points by setting a threshold to filter valid signals that meet the criteria. While fast and effective in low-orbit satellite signal processing, its recognition rate for far-Earth satellites is below 1%.

When the algorithm filtering effect is poor, and there are a large number of noises are mislabeled, the filtering effect can be improved by increasing the comparison point n and the set threshold, but the increase in the value of comparison point n increases the amount of computation of the computer, and the increase in the threshold in the case of low echo rate, the rate of leakage detection will increase [27]. Therefore, the method of multiple iterations is used to improve the filtering effect, [28] proposed a two-filter algorithm strategy, where a second Graz filter is applied after the first, comparing the current point with the previous 300 points. If the threshold is met, the signal is considered valid.The secondary filter adds a small amount of computation but does not affect the real-time nature of the signal recognition and improves the filtering effect, the disadvantage is that it performs poorly in the processing of the satellite data with weak echo signals and has a high rate of miss detection.T.M. Ma combined primary filtering with polynomial fitting, using a first-stage Graz filter to perform linear fitting on the points that meet the filtering criteria. After excluding outliers, a new curve is fitted, and the current point is evaluated by calculating its residual against the new curve. If the residual is below a set threshold, the signal is considered valid. Compared to primary filtering, this approach enhances noise exclusion and improves the recognition of weak signals, However, the false recognition rate is high [29] .Literature [30] proposed a deep learning data processing method, using the residual maps of manually identified signals and those recognized by the GRAZ algorithm as training targets for a deep neural network to identify valid signals. Additionally, an autoencoder was employed to remove noise, which increased the level of automation compared to manual recognition. However, this method has not been applied to range data with significant prediction deviations and, like the GRAZ algorithm, it is unable to identify echo signals from high-orbit satellites. Zequn Lv[31] employs image denoising technology to enhance data processing efficiency within SLR systems. This method identifies valid signal points by binarizing the image and using median filtering for noise removal.The approach significantly improves the accuracy of data recognition; however, its application is limited to the preprocessing stage and does not support real-time recognition of valid signals.

In summary, while many of the aforementioned methods demonstrate good performance, they fall short in achieving real-time signal processing. Techniques such as Poisson filtering and manual screen recognition effectively handle noise but are either too slow or require significant manual intervention. Histogram-based methods, including the Graz algorithm, show excellent results for low-orbit satellites;however their recognition rate for far-Earth satellites is inadequate. Similarly, although polynomial fitting and the two-stage Graz filtering algorithm can process signals in real time, enhancing noise exclusion and weak signal recognition, they exhibit high false and missed detection rates. This hinders the ability of the operators to adjust the laser position in a timely manner, making these methods less effective for practical observation tasks.

To address the challenge of weak echo signals and high noise levels in satellite laser ranging for medium- and high-orbit satellites, which hinders real-time and accurate signal identification, we propose a novel and efficient real-time signal processing method. This method employs a multi-stage filtering strategy, inheriting the speed of the Graz filtering algorithm while integrating a histogram-based secondary filtering approach to improve the recognition rate for medium- and high-orbit satellites. Additionally, we apply the least squares method to fit strong echo signals into a predictive straight line, thereby establishing a predictive model that mitigates the high false detection rate associated with traditional two-stage filtering algorithms.The proposed algorithm was validated using real-world observation data, demonstrating its capability to accurately and reliably identify weak signals in SLR in real-time.The experimental results confirm the effectiveness and robustness of the algorithm.

To clearly demonstrate the performance differences between existing methods and the proposed method, the table below compares various processing methods and highlights the advantages and disadvantages of each, including our new filtering method.

**Reviewer #1:**

**the authors of manuscript "An Efficient Real-Time Signal Processing Method for Satellite Laser Ranging" provided a methor to a kind of real-time SLR data processing method, and solved the challenges brought by the weak echo signal and high noise level of the high-orbit satellite clock. The research method is highly innovative, the result improvement is significant, and the research content conforms to the scope of PLOS ONE.**

Author’s Response: We sincerely thank the reviewer for their positive evaluation of our manuscript "An Efficient Real-Time Signal Processing Method for Satellite Laser Ranging." Your encouraging feedback affirms the value of our work and motivates us to further refine our study.

**Reviewer #2:**

1. **What challenges were encountered when testing the algorithm with real-world data, and how were they addressed?**

Author’s Response:We thank the reviewer for raising this insightful question regarding the challenges encountered during real-world data testing. Below, we detail the specific challenges and the corresponding solutions we implemented.

(Discussion:Challenges and Solutions)

Challenge 1: High Noise Levels in Echo Signals.Real-world data often contain significant noise due to background sky interference, system noise, and weak echo signals, especially for high-orbit satellites and space debris. These high noise levels can result in incorrect identification of noise as valid signals.Solution: The proposed algorithm incorporates a secondary filtering mechanism and polynomial fitting to reduce false detection. By leveraging the histogram-based secondary filtering approach and fitting residual maps, the algorithm effectively identifies valid signals even under noisy conditions.

Challenge 2: Weak Echo Signals from High-Orbit Satellites. Signals from medium- and high-orbit satellites are often weak, making them difficult to distinguish from noise. Traditional methods, such as the Graz algorithm, fail to identify these signals due to their low recognition rate for distant satellites. Solution: The algorithm uses a multi-stage filtering strategy, including least squares fitting to predict signal trajectories. This predictive model helps retain weak signals that might otherwise be excluded by threshold-based methods.

Challenge 3: Large Volume of Data.Real-world data involve processing a massive volume of observations, such as 100,000 data points per 100 seconds, which can significantly increase computation time and make real-time processing challenging.Solution: The algorithm was optimized for efficiency by employing a grid-based filtering mechanism that reduces computational overhead. Additionally, the histogram-based approach partitions data for parallel processing, ensuring faster computation without compromising accuracy. Traditional software displays all raw data and marks valid signals, whereas this study only displays valid signals identified by the algorithm, significantly improving the processing speed.

Challenge 4: Dynamic signals change rapidly.1.Sudden Signal Weakening: In scenarios where signal strength decreases abruptly, such as when observing high-orbit satellites or space debris, the algorithm mitigates the impact of noise by relaxing the thresholds for weak signal identification. The predictive line derived from historical valid signals further aids in recognizing weak signals that might otherwise be missed.2.Sudden Signal Intensification: When the signal intensity unexpectedly increases (e.g., due to improved atmospheric conditions), the processing of strong signal echoes resembles that of low-orbit satellites. This algorithm builds on the Graz filtering algorithm with a second filtering stage. Since the Graz algorithm achieves nearly 100% recognition accuracy for low-orbit satellite signals, the proposed algorithm is theoretically highly effective under such conditions. Experimental results further validate this performance.3.Adaptability to Changes in Signal Frequency:The algorithm operates independently of signal frequency by processing the time-series data sequentially. Adjustments to the time window and grid settings ensure compatibility with varying signal densities, allowing the algorithm to adapt to fluctuations in the frequency of detected echoes.

Challenge 5: Parameter Optimization.Setting optimal parameters (e.g., thresholds, window size, grid dimensions) required careful tuning to balance false detection and missed detection rates.Solution: Parameters were initially set based on values from previous studies and were continuously adjusted during testing with real data to ensure robust performance across various scenarios.

The challenges encountered during real-world data testing primarily revolved around noise, weak signals, computational efficiency, and signal discontinuity. By incorporating adaptive filtering techniques, predictive modeling, and grid-based data partitioning, the proposed algorithm addressed these challenges effectively, achieving high accuracy and robustness in identifying weak echo signals in real-time.

2. **While the paper acknowledges limitations regarding threshold adaptability and the reliance on operator experience, it would benefit from a more in-depth exploration of how these limitations might impact practical applications in various environments.**

Author’s Response:We thank the reviewer for highlighting the need for a more in-depth exploration of how the acknowledged limitations regarding threshold adaptability and reliance on operator experience might impact practical applications in various environments.Your suggestion will make our article more comprehensive and clearly articulated.

(Discussion:Limitations)

Despite these improvements, our method is not without its limitations. The adaptive nature of the threshold settings is crucial for balancing signal detection and noise exclusion. However, in cases where echo signals are extremely weak, even our relaxed threshold settings may result in missed detections. In Addition, while the computational efficiency of our algorithm is improved compared to traditional two-stage filtering methods, further optimization is required to handle larger datasets or more complex observation scenarios.

Another limitation of the proposed algorithm is its lack of strong adaptability. Threshold setting relies on the operator's experience and cannot be automatically adjusted based on the actual signal echo rate.

The setting of parameters such as grid size, sliding window duration, and comparison thresholds often relies on the operator's experience. This dependency introduces variability in algorithm performance, as inexperienced operators may struggle to select optimal values. Threshold settings significantly impact the algorithm's effectiveness: overly low thresholds may result in excessive noise being misidentified as valid signals. In such cases, when the laser fails to hit the target satellite, the resulting echoes, which are purely noise, could be misinterpreted as valid data.Conversely, if the threshold is set too high, the already weak echoes from medium- and high-orbit satellites may be filtered out. This could lead operators to mistakenly believe the laser missed the satellite, prompting unnecessary adjustments to the laser position and hindering observations.

The lack of threshold adaptability and reliance on operator expertise can increase false positives or missed detection, limit the algorithm's scalability, and reduce its level of automation, thereby compromising performance in diverse environments.

**3. What criteria were used to determine the optimal values for the parameters used in your filtering algorithm?**

Author’s Response:We appreciate the reviewer’s question regarding the criteria used to determine the optimal values for the parameters in our filtering algorithm.Reasonable parameter settings are crucial for achieving optimal algorithm performance. We have detailed the basis for parameter selection, and your suggestion adds rigor to our study. We fully agree with your perspective.

(Discussion:Basis for parameter settings)

Criteria for Determining Optimal Parameter Values in the Filtering Algorithm:

1. Accuracy of Signal Identification(Minimizing False and Missed Detections).The primary criterion for parameter optimization was to achieve a low false detection rate (Fr) and a low missed detection rate (Lr).Parameters such as comparison threshold (δ),and minimum valid points (θ) were iteratively adjusted and tested on real-world datasets with varying signal-to-noise ratios. The goal was to balance these two metrics, ensuring accurate identification of weak and valid signals while minimizing noise recognition.

2.Real-Time Processing Efficiency.Ensuring real-time applicability required parameters that minimized computational load while maintaining accuracy. Grid sizes (e.g., large square grid width and height, small square grid step size) and sliding time window duration were tuned to optimize processing time without sacrificing the algorithm's effectiveness. Benchmarks were set based on processing speeds faster than traditional two-stage Graz filtering algorithms.

By combining theoretical insights, real-world data analysis, and iterative tuning, the optimal parameter values were systematically identified to maximize the filtering algorithm’s accuracy, robustness, and real-time applicability.

**4.The paper makes valuable comparisons with traditional algorithms; it would be significantly enhanced by incorporating a wider range of diverse filtering techniques for evaluation.**

Author’s Response: We appreciate the Reviewer's suggestion to incorporate a wider range of filtering techniques for evaluation. However, after conducting an extensive literature review, we found that research on real-time signal processing for satellite laser ranging (SLR) is quite limited. The following points provide a detailed explanation:

Limited Availability of Real-Time SLR Signal Processing Algorithms:

1. The Graz algorithm series remains the most effective and widely recognized approach in the field of SLR real-time signal processing. As an open-source template provided by NASA for global researchers (https://ilrs.gsfc.nasa.gov/technology/software/index.html), it serves as the benchmark for real-time SLR signal processing algorithms. By comparing our proposed algorithm with the Graz algorithm series, we believe we have already demonstrated its superior characteristics and outstanding performance.
2. Beyond the Graz algorithm series, we were able to identify only one other real-time SLR signal processing algorithm[1]. However, its performance is significantly suboptimal, with a missed detection rate approaching 50%. Due to this poor performance, we consider it to be incomparable and irrelevant for meaningful evaluation.

Distinction Between Real-Time and Non-Real-Time Signal Processing:

1. Many other algorithms found in the literature focus on non-real-time signal processing[2-7]( In addition to the algorithms related to the Graz algorithm, which can achieve real-time processing, all the other algorithms mentioned in this paper are used for preprocessing.), which involves processing data after collection. These methods often achieve excellent results by leveraging more complex algorithms.
2. However, real-time signal processing presents unique challenges, as it requires exceptionally fast computation speeds and lower algorithmic complexity to ensure timely results. Non-real-time algorithms, by contrast, are not constrained by such requirements, making comparisons between the two categories inherently unfair and technically uninformative.

Focus of This Study:

Our primary goal is to enhance real-time SLR signal processing. By comparing our algorithm with the Graz algorithm series, the most prominent benchmark in this domain, we have validated its efficiency, robustness, and superiority. We believe this comparison is sufficient to highlight the contributions and advancements presented in our work.

Conclusion:
Given the limited availability of real-time SLR signal processing algorithms and the obvious differences between real-time and non-real-time methods, we respectfully declare that further comparison with a broader range of real-time algorithms is not feasible. Additionally, comparisons with other non-real-time algorithms are irrelevant to the focus of our research.We believe that the current comparisons adequately demonstrate the merits of our proposed algorithm within the appropriate context of real-time SLR signal processing.Thank you for your sincere suggestions. Your input has been very helpful to my article, and we have carefully considered your proposal. We hope you understand that some aspects may not be fully adopted.

[1] Peng Z, ZHANG Y, Xiaoya Z. Echo online extraction for diffuse reflection laser ranging[J]. Acta Optica Sinica, 2015, 35(10): 1028002.
[2] Tong L, Hao C, Ming S. Effective echo extraction for space debris laser ranging using randomized Hough transform[J]. Chinese Journal of Lasers, 2016, 43(4): 0408002.
[3] Yanyu, Liu et al. “Pre-process algorithm for satellite laser ranging data based on curve recognition from points cloud.” Geodesy and Geodynamics 3 (2012): 53-59.
[4] Zhao, Y., Fang, Q., Xie, Y. (2007). An automatic data processing algorithm for Satellite Laser Ranging. In Proceedings of the 2007 Annual Conference of the Metallurgical Automation Branch of the China Metrology Association (pp. 5). Metallurgical Automation Research and Design Institute.
[5] Tang, R. (2015). Laser ranging signal processing and application for space targets. University of Chinese Academy of Sciences.
[6] Rodríguez J, Appleby G, Otsubo T, et al. Assessing and enforcing single-photon returns: Poisson filtering[C]20th international workshop on laser ranging. Potsdam, Germany. https://cddis. nasa. gov/lw20/docs/2016/papers/45-Poisson_paper. pdf. 2016.
[7] Zhang, H., Zhang, Z., Qin, S., et al. (2009). The functions of the station forecast and data preprocessing software for satellite laser ranging at Shanghai Astronomical Observatory. Annual Report of Shanghai Astronomical Observatory, Chinese Academy of Sciences, (00), 153-160.

**5. How does the algorithm perform with dynamic signals that may change rapidly during observations? Is it capable of adapting to sudden changes in signal strength or frequency?**

Author’s Response: We appreciate the reviewer’s insightful question regarding the algorithm’s performance with dynamic signals and its adaptability to sudden changes in signal strength or frequency. Including an explanation of this situation in the article would provide a more comprehensive discussion of the algorithm.

(Discussion:Challenges and Solutions)

1. Sudden Signal Weakening: In scenarios where signal strength decreases abruptly, such as when observing high-orbit satellites or space debris, the algorithm mitigates the impact of noise by relaxing the thresholds for weak signal identification. The predictive line derived from historical valid signals further aids in recognizing weak signals that might otherwise be missed.
2. Sudden Signal Intensification: When the signal intensity unexpectedly increases (e.g., due to improved atmospheric conditions), the processing of strong signal echoes resembles that of low-orbit satellites. This algorithm builds on the Graz filtering algorithm with a second filtering stage. Since the Graz algorithm achieves nearly 100% recognition accuracy for low-orbit satellite signals, the proposed algorithm is theoretically highly effective under such conditions. Experimental results further validate this performance.
3. Adaptability to Changes in Signal Frequency:The algorithm operates independently of signal frequency by processing the time-series data sequentially. Adjustments to the time window and grid settings ensure compatibility with varying signal densities, allowing the algorithm to adapt to fluctuations in the frequency of detected echoes.

The proposed algorithm demonstrates strong adaptability to dynamic signals during observations, effectively managing sudden changes in signal strength or frequency through its multi-stage filtering and predictive modeling strategies. Its real-time processing capabilities ensure robust performance in rapidly changing observation environments, which is critical for satellite laser ranging tasks.
